# Availability and functionality of neonatal care units in healthcare facilities in Mtwara region, Tanzania: The quest for quality of in-patient care for small and sick newborns

**DOI:** 10.1371/journal.pone.0269151

**Published:** 2022-11-21

**Authors:** Serveus Ruyobya Kamala, Zamoyoni Julius, Efraim M. Kosia, Fatuma Manzi

**Affiliations:** 1 Department of Health Systems, Policy, Economic Evaluations, Ifakara Health Institute (IHI), Dar Es Salaam, Tanzania; 2 Department of Global Health and Bio-Medical Sciences (GHBM), School of Life Sciences and Bioengineering, Nelson Mandela African Institution of Science and Technology (NM-AIST), Arusha, Tanzania; 3 Department of Health, Mtwara Regional Secretariat, Mtwara, Tanzania; University of Washington, UNITED STATES

## Abstract

**Background:**

Evidence shows that delivery of prompt and appropriate in-patient newborn care (IPNC) through health facility (HF)-based neonatal care and stabilization units (NCU/NSUs) reduce preventable newborn mortalities (NMs). This study investigated the HFs for availability and performance of NCU/NSUs in providing quality IPNC, and explored factors influencing the observed performance outcomes in Mtwara region, Tanzania.

**Methods:**

A cross-sectional study was conducted using a follow-up explanatory mixed-methods approach. HF-based records and characteristics allowing for delivery of quality IPNC were reviewed first to establish the overall HF performance. The review findings were clarified by healthcare staff and managers through in-depth interviews (IDIs) and focus group discussions (FGDs).

**Results:**

About 70.6% (12/17) of surveyed HFs had at least one NCU/NSU room dedicated for delivery of IPNC but none had a fully established NCUs/NSU, and 74.7% (3,600/4,819) of needy newborns were admitted/transferred in for management. Essential medicines such as tetracycline eye ointment were unavailable in 75% (3/4) of the district hospitals (DHs). A disparity existed between the availability and functioning of equipment including infant radiant warmers (92% vs 73%). Governance, support from implementing patterns (IPs), and access to healthcare commodities were identified from qualitative inquiries as factors influencing the establishment and running of NCUs/NSUs at the HFs in Mtwara region, Tanzania.

**Conclusion:**

Despite the positive progress, the establishment and performance of NCUs/NSUs in providing quality IPNC in HFs in Mtwara region is lagging behind the Tanzania neonatal care guideline requirements, particularly after the IPs of newborn health interventions completed their terms in 2016. This study suggests additional improvement plans for Mtwara region and other comparable settings to optimize the provision of quality IPNC and lower avoidable NMs.

## Introduction

Neonates are at the highest risk of morbidity and mortality throughout their first 28 days of life, and an estimated 2.5 million neonates globally died during this vulnerable time in 2017/2018 [1, 2] About ¼ of all global NMs occur within the first 24 hours before post-delivery discharge, and ¾ occur within the first week of birth [3, 4].

Studies have shown that NMs account for the majority of global under-five mortalities (U5Ms) and that, the proportion has risen in tandem with the fall in NMs, with NMs contributing 47% to the U5Ms in 2020 [5] versus 40% in the 1990s [4, 5]. Since the 1990s, NMs have often lagged behind U5Ms in terms of reduction. Whereas U5Ms declined by 47% between 2000 and 2016, NMs fell by just 39% during the same period [5]. Tanzania achieved the millennium development goal (MDG-4) in the reduction of U5Ms and infant mortalities (IMs) but failed to achieve the MDG-5 in reduction of NMs and maternal mortalities (MMs) [6]. Between 1999 and 2015, U5Ms and IMs in Tanzania had fallen by 54% and 57% respectively, while NMs remained the same at 26 deaths per 1000 live births between 2004 and 2016 [6].

Recent studies have revealed a significant global decline in NMs from 37 deaths per 1000 live births in the 1990s to 17 deaths per 1,000 live births in 2020 [5]. However, during the same period, NMs remained unacceptably high in low-and middle-income countries [7, 8]. Sub-Saharan Africa (SSA) and Southern Asia (SA) accounted for 79% of the total GNMs in 2017 [8], with SA sharing 39% followed by SSA sharing 38% [9]. About 23% of NMs occurring in SSA were reported in West and Central Africa [8]. Nigeria, Ethiopia, and Tanzania are the countries currently with the highest NMs in SSA [10].

Tanzania was among the top 10 countries with the most NMs worldwide, with 43, 000 neonates dying in 2019 [4]. The population-based surveys have also indicated that NMs in Tanzania ranged between 26 and 40 deaths per 1000 live births during the last three decades, with wide variations between regions and councils [11–13].

Prematurity and low birth weight (LBW) complications, neonatal infections, intrapartum-related complications, congenital anomalies [3], absence of quality care [14–16], and/or lack of healthcare at all [16] are the most reported leading causes of GNMs. Similar causes of NMs have been reported in Tanzania [17–19].

About 75% of global NMs of all causes can be prevented [20], however, this requires a combination of health system approaches along the continuum of care [21] such as increasing access to high-quality antenatal care, skilled birth care, postnatal care for mother and baby [4, 20, 22], emergence and routine care at birth [21, 22], and provision of high-quality IPNC services [16, 23–25].

Evidenced shows that, availability and proper functioning of NCUs/NSUs at the HFs ensure delivery of IPNC by providing an avenue for sick and small newborns to be admitted, diagnosed, and effectively managed [26, 27]. It is estimated that 20% of all NMs can be averted through interventions to small and sick newborns implemented in NCUs/NSUs [28]. Such interventions include thermal protection, feeding and breathing support; treatment of jaundice; prevention and treatment of infection, mechanical ventilation, and provision of intermittent positive-pressure therapy or surgery.

Regarding the Tanzania neonatal care guideline [29], all health centers (HCs) are mandated to have fully functional NSUs for the care of moderately sick and small newborns. Complicated newborn cases at the HCs should promptly be referred to the hospitals for more specialized care in NCUs. An NSU is regarded as fully functional upon availability of a general neonatal ward (GNW) for the care of sick but stable newborns, an isolation room for contagious newborn cases, and a kangaroo mother care (KMC) room for care of small newborns, while a fully functional NCU requires all provisions at the HC with the addition of a high dependence unit (HDU) at DHs and a neonatal intensive care unit (NICU) at regional and tertiary hospitals for care of more sick and small newborns. As a result, regular tracking and/or monitoring of HF’s progress in establishment and performance of NCUs/NSUs in the provision of quality IPNC regarding the available guidelines, as well as understanding the factors influencing such progress, is important for a healthcare systems resilience.

Health facilities’ performance in the provision of emergent obstetric and newborn care (EmONC) has been measured and/or tracked in several studies [26, 30] but the focus has primarily been on obstetric care [23, 24]. In most healthcare settings, the HFs’ performance in the delivery of quality IPNC is currently inadequately monitored [24]. However, even few studies that have examined HFs’ performance in the delivery of quality IPNC [31] have only focused on structural aspects and not on understanding factors influencing such performance.

Therefore, this study evaluated HFs for the availability and performance of NCU/NSUs in the provision of quality IPNC, along with an exploration of factors influencing such performance to inform continuous improvement plans to optimize the reduction of preventable NMs.

## Materials and methods

### The study settings

The study was conducted in Mtwara region in Tanzania because, between 2010 and 2015, NMs in Mtwara region was higher (47 deaths per 1000 live births) above the national average of 26 deaths per 1000 live births [32]. About 17 HFs that met inclusion criteria (operating for not less than three years and also providing EmONC services) from among 36 HFs mandated to deliver IPNC were included in this study. Of all HFs included in the study, 76.5% (13/17) were publicly owned while 23.5% (4/17) were non-public. The majority 64.7% (11/17) of study HFs were HCs, and 35.3% (6/17) were hospitals. Mtwara region is bordered on the north by the Lindi region, on the East by the Indian Ocean, on the South by Mozambique, and on the West by the Ruvuma region. It has a population of approximately 1,507,426 people (according to 2021 projections of the 2012 national census).

### Study design

**Fig 1:**
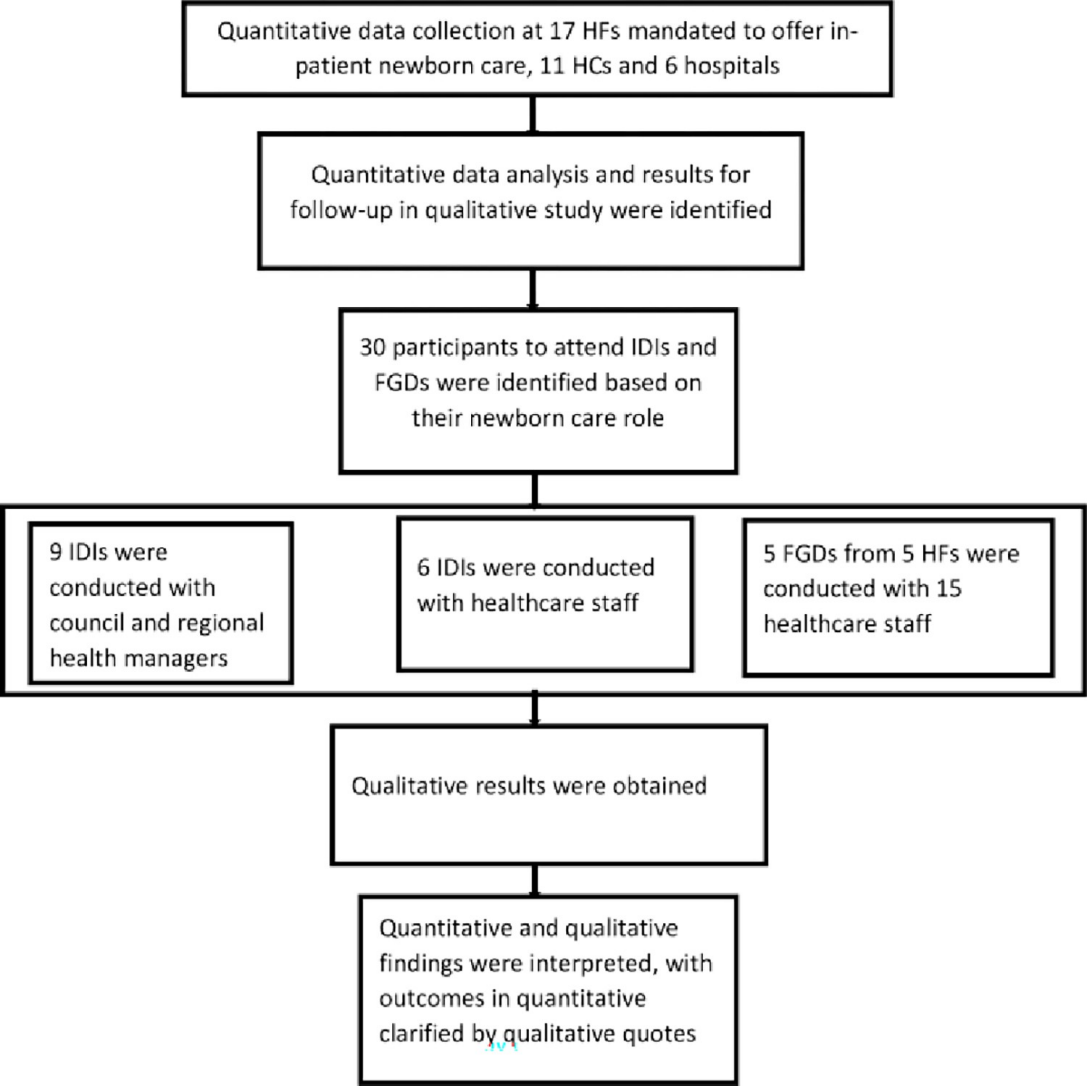
Flow chart of the study design.

A cross-sectional study was conducted using a follow-up explanatory mixed-methods approach (Fig 1).

**Fig 2:**
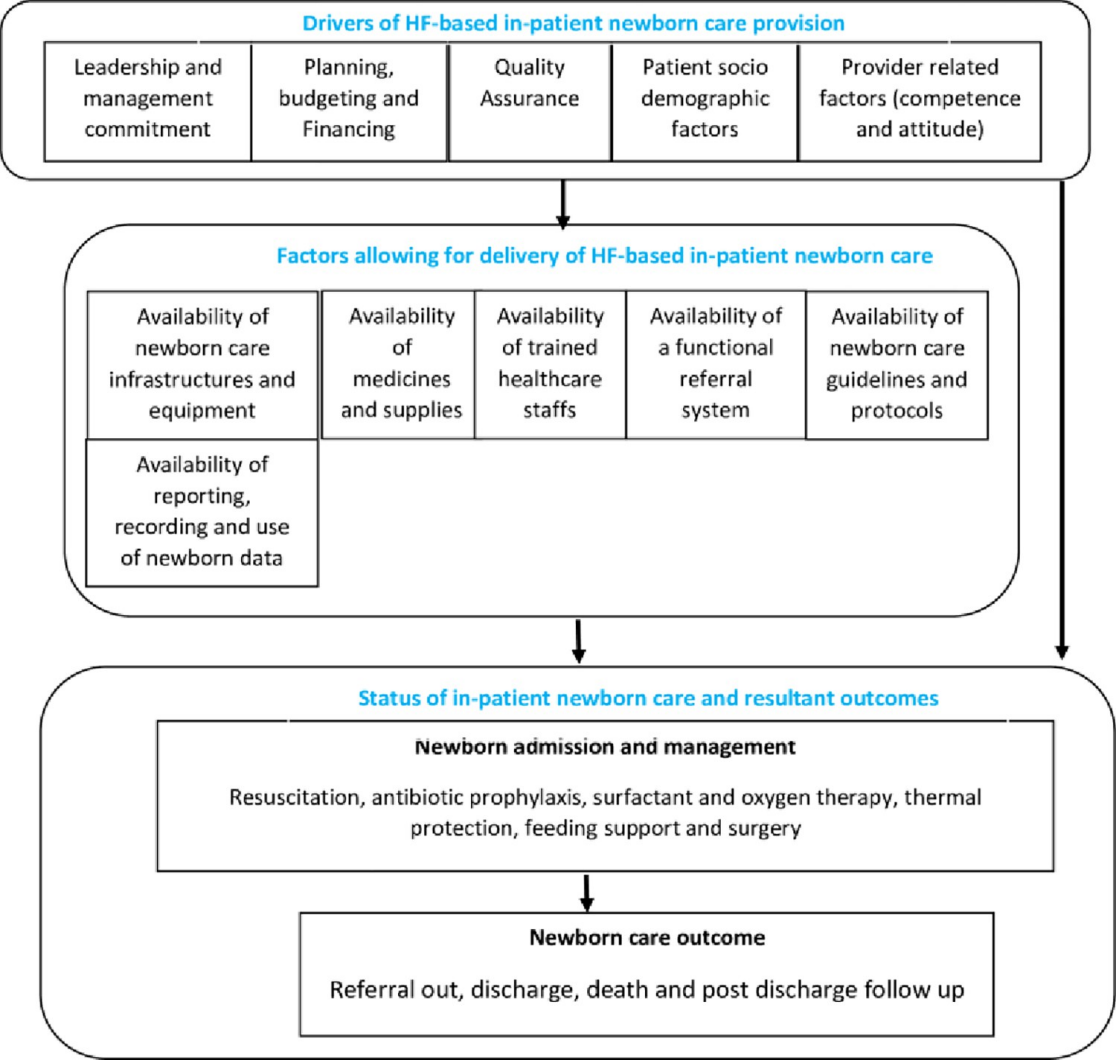
Framework for maternal and newborn care quality.

Quantitative data was collected and analyzed first to establish the overall HF status on availability and functionality of NCUs/NSUs followed by qualitative data collection and analysis that obtained clarifications of the quantitative findings [33]. Quantitative data was collected on HF-based newborn records and characteristics that allow for the delivery of quality IPNC such as; newborn birth, admission, management h records, the availability and functioning of newborn care infrastructure and equipment, the availability of medicines and supplies, referral systems, recording and reporting systems, and evidence of newborn data use in decision making as well as healthcare staff training on in-patient newborn care. The investigation was guided by a framework for maternal and newborn care quality [27, 34] as indicated in (Fig 2).

### Data collection

The data was collected in three ways: 1) review of newborn records updated in health management information system (HMIS) and newborn registers from January to December 2020, 2) health facility-based assessment of factors for delivery of IPNC services, and 3) interviews (IDIs and FGDs) with healthcare staff and managers to clarify the review and assessment findings in 1 and 2. In the HF-based assessment, a standardized electronic checklist embedded in the tablets was used after being piloted at two (2) non-study facilities for validation and improvement. Indicators for facility delivery of quality IPNC included in the checklist were extracted from the survive and thrive report [16], WHO-Early Essential Newborn Care (EENC) guideline [25], Tanzania neonatal care guideline [29], the WHO standards for improving quality of maternal and newborn care in HFs [27], and the UNICEF tool kit for settling up special NCUs and NSUs [35]. The HF-based assessment was done through an observational and interview schedule. Two field investigators with prior experience in public health research were oriented for one day to conduct HF assessment and interviews. The data collection process was supervised by the principal investigator between June and August 2021.

After preliminary analysis of quantitative data, outcomes within each HF indicator for delivery of quality IPNC were clarified through FGDs and IDIs. The IDIs were conducted with regional and council health managers having experience in newborn care and with some healthcare staff (midwives, nurses, and doctors) working in NCUs/NSUs at HFs with insufficient staff, while FGD only involved similar healthcare staff at HFs that had suitable numbers of staff.

### Data management and analysis

After being validated for completeness, quantitative data was exported to Stata version 14 for statistical analysis. Proportional means and overall scores were used to summarize the results, which were then displayed in tables for comparison. The primary outcome of the analysis was an HFs’ availability and functionality of NCUs/NSUs. Recording audios for IDIs and FGDs from representative HFs (8 with and 4 without any form of NCU/NSU) were transcribed, read, and analyzed manually by 4 authors of this work. Contents describing each quantitative finding were manually summarized on a Microsoft Excel spreadsheet based on the similarity of responses from interviewees. Only important quotes from contents summarized in each quantitative finding were translated into the English language for reporting.

### Ethical approval and consent to participate

Ethical approval for this study was obtained from the Ifakara Health Institute Ethical Review Board with permit **No. IHI/IRB/No.22-2021**. All participants provided written informed consent before they participated in the interviews. A letter introducing investigators to the study HFs was obtained at a respective administrative council.

## Results

### Quantitative findings

The presentation of quantitative findings falls under eight areas as follows;

**1) Availability of neonatal care and stabilization units.** About 29.4% (5/17) of assessed HFs, all of which were HCs had no NSUs indicating that needy newborns attended there were either being referred out, admitted to non-newborn wards, and/or returned home. Out of 70.6% (12/17) of HFs that had at least any NCU/NSU room dedicated to delivering IPNC services, only 75.0% (9/12) had rooms located inside or close to the maternity ward to avoid delays in newborn care during need (Table 1).

**Table 1:** Availability of NCU/NSUs at the HFs (N = 17).

Variable	Area/Parameter	Outcome (Frequency), n (%)
**Availability of NCU/NSU room**	Regional Hospitals	2 (100.0)
	District Hospitals	4 (100.0)
	Health centers	6 (54.5)
**Location of NCU/NSU within the HF**	Nearby labor/maternity block	9 (75.0)
	Accessible by ambulance	11 (91.7)
**Availability of important NCU/NSU clinical spaces**	Kangaroo Mother Care room	7 (58.3)
	General neonatal ward	8 (66.7)
	Isolation room	1 (16.7)
	High Dependency Unit/Neonatal Intensive Care Unit	3 (50.0)

**2) Newborn cases and in-patient care provision at the HFs.** Prematurity and low birth weight complications accounted for health risks to 33.3% (1,606/4,819) newborns, followed by 32.1% (1,545/4,819) newborns whose mothers were absent (died or unconscious) as a result of complicated labor. Unknown causes accounted for health risks to 34.8% (1676/4891) of newborns. Only 74.7% (3,600/4,819) of newborns at risk were admitted and/or transferred to NCUs for management. Newborn admissions were fewer at HCs 21.9% (169/770). About 98.3% (1578/1606) of small newborns in NCUs/NSUs did not receive early infancy vaccinations (with OPV and BCG) against poliomyelitis and tuberculosis (Table 2).

**Table 2:** Newborn service provision status by HF level HF-based registers.

Area/Variable		Outcome (Frequency), n (%)			
		Regional Hospital	District Hospitals	Health centers	Overall
**A. Newborn data**	Live birth	2,733	7,814	4,231	14,778
**B. Newborns at risk (B/A)**		1,196 (43.8)	2,853 (36.5)	770 (18.2)	4,819 (32.6)
**C. Risk factors (C/B)**	Prematurity	241 (20.2)	789 (27.7)	576 (74.8)	1606 (33.3)
	Birth asphyxia	129 (10.8)	384 (13.5)	11 (1.4)	524 (10.9)
	Sepsis	169 (14.1)	507 (17.8)	11 (1.4)	687 (14.3)
	Mother absent	592 (38.7)	917 (32.1)	165 (21.4)	1545 (32.1)
	Others	273 (22.8)	795 (27.9)	608 (79.9)	1676 (34.8)
**D. Newborn admission (D/B)**	Admission to NCUs/NSUs	1117 (93.4)	2314 (81.2)	169 (21.9)	3600 (74.7)
**E. Vaccination**	Small newborns vaccination	2 (0.8)	21 (2.7)	5 (0.9)	28 (1.7)
**F. Newborn outcomes (E/D)**	Discharged alive	586 (52.5)	1718 (74.2)	47 (27.8)	2351(65.3)
	Death in NCU	63 (5.6)	97 (4.2)	0	160 (4.4)
	Referral out	12 (0.3)	13 (0.4)	42 (24.9)	67 (1.9)
	Unknown status	456 (40.8)	486 (21.0)	80 (47.3)	1022 (28.4)

**3) Healthcare staff training on newborn care.** Out of 76 healthcare staff who engaged in newborn care during the day of assessment in 12 HFs, only 24 (31.6%) were trained. A major training gap of 4.5% (1/22) existed among healthcare staff engaging in newborn care at HCs (Table 3).

**Table 3:** Status of health care staff training on in-patient newborn care.

Facility level	Outcome (Frequency), n (%)	
	Healthcare staff engaged in special newborn care	Healthcare staff trained
**Regional Hospitals**	22 (100.0)	5 (22.8)
**District Hospitals**	32 (100.0)	18 (56.3)
**Health centers**	22 (100.0)	1 (4.5)
**Total**	**76 (100.0)**	**24 (31.6)**

**4) Availability of essential medicines and medical supplies.** Essential medicines like tetracycline eye ointment, Phenytoin, and vitamin K were only available in fewer HFs (Table 4).

**Table 4:** Availability of essential medicines by the level of newborn care, (N = 12).

Medicine category	Medicine Item	Regional Hospital	District Hospitals	Health Centre	Overall score
**Antibiotics**	Gentamicin	2 (100.0)	4 (100.0)	6 (100.0)	12 (100.0)
	Cefotaxim	2 (100.0)	4 (100.0)	n/a	6 (100.0)
	Vancomycin	2 (100.0)	n/a	n/a	2 (100.0)
	Imipenem	2 (100.0)	n/a	n/a	2 (100.0)
	Levetiracetam	2 (100.0)	4 (100.0)	n/a	6 (100.0)
	Midazolam	2 (100.0)	n/a	n/a	2 (100.0)
	Nevirapine	2 (100.0)	4 (100.0)	6 (100.0)	12 (100.0)
**Anticonvulsants**	ABIDEC Multivitamin	2 (100.0)	4 (100.0)	6 (100.0)	12 (100.0)
	Ceftriaxone	2 (100.0)	4 (100.0)	5 (83.3)	11 (91.7)
	Ampicillin	2 (100.0)	4 (100.0)	4 (676.7)	10 (83.3)
	Metronidazole	2 (100.0)	3 (75.0)	5 (83.3)	10 (83.3)
**Prophylaxis**	Isoniazid	2 (100.0)	3 (75.0)	5 (83.3)	10 (83.3)
	Zidovudine	2 (100.0)	3 (75.0)	4 (676.7)	9 (75.0)
	Phenobarbitone	2 (100.0)	3 (75.0)	3 (50.0)	8 (66.7)
	Vitamin K	2 (100.0)	1 (25.0)	2 (33.0)	5 (41.7)
	Phenytoin	1 (50.0)	1 (25.0)	n/a	2 (33.3)
	Tetracycline eye ointment	2 (100.0)	1 (25.0)	6 (100.0)	11 (91.7)

All basic supplies and consumables were found in more than two-thirds of surveyed HFs during the day of the assessment, except for the feeding cups and baby diapers which were found in less than 50% of the HFs. Infusion cups were reported to be used instead of feeding cups in those facilities that had no feeding cups (Table 5).

**Table 5:** Status of essential supplies at HFs with NCU/NSU, (N = 12).

Supplies Items	Outcome (Frequency), n (%)			
	Regional Hospitals	District Hospital	Health centers	Overall availability
**Oxygen and oxygen tube**	2 (100)	4 (100)	4 (66.7)	10 (83.3)
**Sterile gloves**	2 (100)	2 (50.0)	4 (66.7)	8 (66.7)
**Disposable syringes (2, 5, 10 ml) and needles**	2 (100)	4 (100)	6 (100)	12 (100)
**Plaster**	2 (100)	4 (100)	6 (100)	12 (100)
**IV cannulas (G24)**	2 (100)	4 (100)	6 (100)	12 (100)
**Feeding cups**	0	1 (25.0)	1 (16.7)	2 (16.7)
**Nasogastric tube**	2 (100)	3 (75.0)	3 (50.0)	8 (66.7)
**Antiseptic solution**	2 (100)	4 (100)	6 (100)	12 (100)
**Swabs**	2 (100)	4 (100)	6 (100)	12 (100)
**Diapers**	1 (50.0)	2 (50.0)	2 (33.3)	5 (41.7)
**Liquid soap for handwashing**	2 (100)	4 (100)	6 (100)	12 (100)
**Burette (a neonatal infusion set)**	2 (100)	1 (25.0)	4 (66.7)	7 (58.8)
**Bedsheets**	2 (100)	3 (75.0)	6 (100)	11 (91.6)
**IV fluids**	2 (100)	4 (100)	6 (100)	12 (100)
**Water for injection**	2 (100)	4 (100)	6 (100)	12 (100)
**Urinary catheters (5 Fr)**	2 (100)	0	4 (66.7)	6 (50.0)
**Cord clamps**	2 (100)	4 (100)	6 (100)	12 (100)
**Glucose strips**	2 (100)	2 (50.0)	4 (66.7)	8 (66.7)
**Lancets**	2 (100)	3 (75.0)	5 (83.3)	10 (83.3)
**Blood sample tubes**	2 (100)	4 (100)	6 (100)	12 (100)
**Burette (a neonatal infusion set)**	2 (100)	1 (25.0)	4 (66.7)	7 (58.3)
**Blood giving set**	2 (100)	3 (75.0)	5 (83.3)	10 (83.3)
**Nasal prongs for oxygen administration (neonatal size)**	2 (100)	3 (75.0)	2 (33.3)	7 (58.3)
**Suction catheters (6, 8, 10 Fr)**	2 (100)	3 (75.0)	4 (66.7)	9 (75.0)

**5) Status of essential newborn equipment and repair plan.** Small oxygen cylinders for transportation of oxygen-dependent patients were lacking in 50.0% (6/12) of surveyed HFs. A variability existed between the availability and functioning of infant radiant warmers 91.6% (11/12) vs 72.7% (8/11) and oxygen concentrators 75.0% (9/12) vs 66.7% (6/9). About 91.6% (11/12) of HFs surveyed had no equipment maintenance and repair plan for the impaired equipment in NCUs/NSUs (Table 6).

**Table 6:** Availability of essential newborn care equipment and repair plan, (N = 12).

Equipment Item	No. of HFs, n (%)	
	Available	Functional
**Resuscitation table**	9 (75.0)	8 (88.9)
**Oxygen concentrator**	9 (75.0)	6 (66.7)
**Suction machine**	9 (75.0)	8 (88.9)
**AMBU bag and mask size**	12 (100)	12 (100)
**Pulse oximeter**	12 (100)	12 (100)
**Penguin suckers**	11 (91.6)	11 (100)
**Digital thermometer**	9 (75.0)	9 (100)
**Measuring tape**	11(91.7)	10 (90.9)
**Bedside lamp for a procedure**	10 (83.3)	9 (90.0)
**Infant radiant warmer/incubator**	11 (91.6)	8 (72.7)
**Small oxygen cylinder for referral**	6 (35.3)	5 (83.3)
**Glucometer (at least one)**	8 (66.7)	8 (100)
**Equipment maintenance and repair plan**	1 (8.3)	1 (100)

**6) The availability of a functional referral system.** About 76.5% (13/17) of assessed HFs (both with and without NCU/NSU) had an ambulance, 11.8% (2/17) HFs had no ambulance but could access a functional ambulance or any vehicle during need, and 11.8% (2/17) HFs had no ambulance or means to access an ambulance or any vehicle during need. Referral books were inadequately available (Table 7).

**Table 7:** Status of the referral system (N = 17).

Referral parameter	Outcomes, n (%)			
	Health Centers	District Hospitals	Regional Hospitals	Overall
**Functional ambulance/vehicle available**	7 (63.6)	4 (100)	2 (100)	13 (76.5)
**No ambulance but have access to any**	2 (18.2)	0	0	2 (11.8)
**No ambulance and no access to any during need**	2 (18.2)	0	0	2 (11.8)
**Fuel for ambulance available (assessment day)**	5 (71.4)	4 (100)	2 (100)	11 (64.7)
**Accessibility of NCU/NSU by ambulance**	6 (100)	4 (100)	1 (50.0)	11 (64.7)
**A mechanism of phone call not on HCP’s cost**	11 (100)	4 (100)	2 (100)	17 (100)
**Payment of benefits to HCPs accompanying the patient during the referral**	7 (63.6)	3 (75.0)	2 (100)	12 (70.6)
**Availability of referral books/forms**	6 (54.5)	2 (50.0)	2 (100)	10 (58.8)

**7) Recording, reporting, and newborn data use.** The study retrieved about 17,136 deliveries and 406 deaths from HMIS reports updated from January to December 2021 as compared to 14,778 deliveries and 160 deaths retrieved from facility registers updated during the same period. The mismatch in newborn data updated in HMIS and registers, and the unavailability of records for care outcome of 28.4% (1022/3600) newborns who were admitted in NCU/NSU (Table 2) indicated inadequate recording and/or reporting. Newborn data recording and reporting tools were available at the majority of HFs **(Table 8)**. There was no proof of newborn death reviews at 64.7% (11/17) of HFs.

**Table 8:** Status of recording, reporting, and neonatal care guidelines, (N = 17).

Area	Variable	No. of HFs, n (%)
**Availability of recording and reporting tools**	Delivery books	12 (70.6)
	Admission and management books	12 (70.6)
	Newborn death register books	12 (70.6)
	A computer to store health data	12 (70.6)
**Availability of recording and reporting tools/facilities**	Access to any source of internet	12 (70.6)
	HMIS focal person	12 (70.6)
	Newborn monthly data updated in HMIS	11 (64.7)
**Use of newborn data for quality improvement**	Regular reviews of new-born deaths	11 (64.7)
	Facilities reviewing newborn deaths	11 (64.7)
**Availability of guidelines and protocols**	Infection Prevention and Control	11 (64.7)
	National Newborn Care Guideline (2019)	9 (52.9)
	Kangaroo Mother Care	4 (23.5)
	Hand washing protocols displayed above hand washbasins	10 (58.8)

**8) Availability of important newborn care guidelines and protocols.** A KMC guideline was unavailable at 76.5% (13/17) of HFs and hand washing protocols were displayed above hand washing basins at 58.8% (10/17) of HFs (Table 8).

### Qualitative findings

We conducted 15 IDIs and 5 FGDs with the council and regional health managers, and healthcare staff (Table 9).

**Table 9:** Interviewed participants and their codes.

Category	Participants	Codes	Number
**Health Managers**	IDI with Zonal health manager	ZM	1
	IDI with the Regional health manager	RM	1
	IDI with Council health manager	CM	7
**Providers**	6 IDIs with midwives, nurses, and doctors	CP	6
	5 FGDs with midwives, nurses, and doctors	FGD	15
**Total**			**30**

### Factors for establishment and running of neonatal care and stabilization units

#### Partnership and collaboration

Analysis of qualitative response identified support from implementing partners as an influence on the establishment and smooth running of NCUs/NSUs at the HFs:

“*Between 2008 and 2015*, *implementing partners (voluntary services overseers* (VSO) *and Deutsche Gesellschaft für Internationale Zusammenar beit* (GIZ)) *supported the establishment and strengthening of NCU services in three district hospitals in Mtwara*. *They procured equipment and medicines*, *and trained healthcare staff on newborn care"* (ZM).

"*Our facility has NCU because VSO supported its establishment*. *Relying on our resources could have been a challenge"* (CM6).

#### Governance and financing-related issues—planning, budgeting, and financing

Priority setting and political commitment were major drivers for the delayed establishment of NCUs/NSUs. Whenever funding was planned for the construction of newborn units, implementation was reported to take place. The stakeholders were quoted as saying: -

"*The construction of facilities comes with government priorities and procedures; you can’t decide to establish NCU if the government has planned for construction of other wards or a theater"* (CM7).

“*Setting up an NCU entails starting little by little*, *first by designating one room and gradually upgrading it*. *For example*, *a CHMT in one council in Mtwara was able to establish NSU by first persuading councilors on the pros and cons of NSU*. *The councilors agreed and dispatched their healthcare staff for clinical attachments*. *As a result*, *one room within the facility was vacated to allow the establishment of NSU*, *and they managed”* (RM).

Failure to plan and budget for the construction of NCUs/NSUs in the annual health plans was stated to slow the establishment and running of NCUs/NSU at the HFs in Mtwara:

"*The ease for the establishment of NCU in our council stems from budgeting on council health plans [Comprehensive Council Health Plans]*. *We are now implementing our plan for the construction of OPD*, *the laboratory*, *and an emergency department and gradually we will construct NCUs as per our strategic plans* (CM1).

"*Our health budget is insufficient*, *which is why establishing NCUs is difficult; but*, *if we had more funds*, *we would have established NCUs*, *particularly in all HCs providing CEmONC services"* (CM2).

Where there were old buildings, the design did not provide room for NCU/NSUs establishment. Participants had the following to say;

"*The existing buildings did not account for the NCU/NSU establishment as they have not enough space to accommodate NCU/NSU; what is being done for the available NCU/NSU is an improvisation"* (RM).

#### Reasons for suboptimal newborn care—community-related barriers

The patients’ socio-demographic factors were identified as major factors delaying care-seeking during newborn illness episodes hence leading to poor newborn health outcomes. These included poverty, socio-cultural norms, past care experience, community attitude toward newborn survival, and maternal awareness of newborn danger signs. The community-related factors are detailed as follows;

**Poverty**The avoidance of transport costs and parents’ inability to bear hospital expenditures encourage home delivery and referral refusal.

"*A caretaker may refuse to take a newborn referral by insisting they don’t have money to afford indirect costs*. *They request for discharge of unwell baby"* (CP6).

"*Sometimes treatment of more premature babies being admitted at the HF for an extended period becomes a challenge when caretakers insist on discharging their newborns before they have graduated from care*, *for the pretext that they are unable to cover the expenditures of their stay"* (FGD2).

#### Socio-cultural norms

Participants highlighted traditional medications and community beliefs as accelerators of NMs. These variables have encouraged home delivery and/or getting late at the facility for delivery;

"*In some communities*, *pregnant mothers do not come for facility delivery until they have drunk or applied some drugs on their wombs believing that such drugs enable them to give birth right away to limit hospital stays*. *At the time they get to the hospital*, *it is either the cervix is yet open or the contractions are too strong*, *which may result in bad newborn outcomes"* (CP3).

"*Pregnant women who have given birth more than once are being convinced that they have mastered labor and delivery*, *therefore encouraged to give birth at home or get late at the facility after they have developed complications"* (FGD3).

#### Community perception- hospital stay and social value of newborns

Given past care experiences in the community, participants indicated extended hospital stays and fear of cesarean section as reasons for late or avoided institutional delivery:

“*When mothers giving birth at home are inquired to state the reasons for home delivery*, *they would tell you that they avoid cesarean section or remaining at the HF for extremely longer”* (FGD3).

A KI stated that placing more value on maternal than newborn survival drives communities to commit less time and resources to seek healthcare during newborn illness. The situation was blamed as being responsible for decelerating regional NM reduction interventions;

"*Newborn and MMs can be reduced by implementing the* ***“Jiongeze Tuwavushe Salama campaign”***. *Unfortunately*, *communities are much more concerned with MM than with NMs”* (RM).

#### Reasons for suboptimal newborn care–health provider-related barriers

The qualitative inquiry revealed various reasons that contributed to poor quality newborn care in NCU/NSU as described below: -

**Healthcare staff competence**Participants of IDIs/FGDs highlighted a lack of newborn care skills among healthcare staff as a barrier to IPNC provision in NCU/NSUs;

“*The main cause of NMs is birth asphyxia*, *which is caused by provider’s inability to monitor labor and identify early newborn danger signs for immediate clinical action”* (CM1).

"*The HCPs in lower-level facilities have no newborn care training*, *as a result*, *they are unable to diagnose newborn’s danger signs promptly*, *resulting in delayed referrals and the resultant bad outcome"* (FGD1).

It was also stated that some sick and small newborns are not sent to NCU/NSUs because providers are unable to sort them as normal or having abnormal clinical conditions;

"*Some needy newborns are not sent to the NCU for management because of poor providers’ comprehension of why the newborn should be sent to the NCU”* (CM1).

The reason for not vaccinating LBWs in NCUs with BCG and OPV vaccines was also stated to be due to worker’s awareness and attitudes;

"*The lack of linkage between the labor ward and RCH units*, *and the provider’s notion that neonates are only vaccinated when they are 2*.*5 kg and above are the reasons why premature and low birth weight babies in NCUs are not vaccinated"* (ZM).

"*Communication barriers between the healthcare staff in NCU/NSU and the RCH unit is the reason LBW babies in NCU/NSUs are not vaccinated*. *The HCPs think that linking newborns in NCU to the RCH unit is not their responsibility"* (CM1).

#### Shortage of newborn care infrastructure and resources

The absence of NCU/NSU at the HFs was a reason highlighted as to why needy newborns were managed locally by being admitted to non-newborn wards or being referred: -

"*Severe birth asphyxia and premature newborn cases are always referred out*, *while those with jaundice or septicemia are retained for care within the maternity or pediatric ward"* (CP2).

"*If I have premature babies that can’t be referred to higher-level hospital*, *I manage them locally*. *I fill bottles with warm water*, *roll in towels and wrap them around the baby and regularly monitor temperature"* (CP2).

Participants highlighted equipment shortage as an obstacle to quality IPNC at the HF:

"*Facility shortage of equipment delays newborn treatment*. *When needy newborns outweigh the available equipment*, *providers prioritize the critically ill"* (FGD3).

"*At the HF*, *limited oxygen concentrators are being rotated among needy newborns time by time from a mildly to severely ill until all are saved"* (RM).

#### Stock-out of medicines and equipment

Participants highlighted that they always face stock-out of newborn lifesaving items specifically in remote health centers due for several reasons. First, when orders are submitted to the medical stores department (MSD), many times newborn related items are unavailable and hence not supplied to the HFs. Second, due to the low caseload of neonates, providers tend to ignore ordering enough stock of medicines to address newborn health problems. Third, due to limited resources, health workers fear ordering newborn supplies and equipment that have a high price. The following quotes present the various issues related to stock-out: -

“*Sometimes MSD is being stocked out by some newborn health commodities upon our request*. *Seeking alternative suppliers takes a long process"* (CM6).

"*Some medicines are unavailable because we only attend to a few newborn cases*, *as a result*, *buying adequate medicines would lead to expiration*. *Ampicillin*, *gentamicin*, *and phenobarbitone are medicines adequately available because are used by persons of all ages"* (CP5).

"*The equipment and medicines are inadequately available at the HF because providers do order them from MSD"* (CM3).

“*Bedside lamps are too costly for a young institution like ours to afford"* (CP2)

#### Poor handling and management of equipment

Poor equipment handling and management failure of controlling institutional resources and facilities were stated as reasons for equipment impairment resulting in a shortage of equipment at the HFs;

"*Misuse and poor equipment handling result in equipment shortage*. *For example*, *Penguin suckers are damaged by being boiled or soaked in bleach [Jic]"* (CM3).

"*The management is not taking good control of resources*. *They just wait until supervisors from the region notice and demand equipment repairs or replacement*. *For instance*, *we once found a plastered [repaired] penguin sucker at the HF*, *we shouted till it was replaced the next day"* (RM).

“*The management commit funds planned for facility preventive maintenance more on the vehicle than on equipment*" (ZM)

The paucity of biomedical technicians was stated as the reason for delayed equipment maintenance and repair;

"*Equipment is not being maintained due to shortage or lack of biomedical technicians*. *However*, *even when technicians are available*, *do not keep an eye on equipment to detect faults; instead*, *they wait to be summoned”* (ZM).

#### Suboptimal recording and reporting of newborn data

Participants linked poor newborn data recording and reporting to a healthcare staff shortage, attitude, human errors, and computer illiteracy;

"*Sometimes healthcare providers are overwhelmed with clinical duties*, *ending up with undocumented work"* (CP4).

"*Poor recording and reporting of newborn information are caused by several reasons including provider’s mindset*. *Providers understand are required to record and report but you find they don’t do"* (CM7).

"*Sometimes reporting newborn data in the electronic system* (HMIS) *is hindered by computer illiteracy*” (RM).

The analysis of qualitative data indicated regular data quality checks as a major enabler of quality recording and reporting of newborn data:

"*Reporting issues do happen in my council*, *but we conduct a quarterly data quality check (DQC) to compare data on HMIS and the registers*. *Furthermore*, *the occurrence of newborn death is promptly reported on HMIS as opposed to cumulating reports till the end of the month"* (CM2).

System bottlenecks were also stated to hinder newborn data reporting into the HMIS:

“*Sometimes the system malfunctions resulting in entry errors"* (RM).

“*Occasionally*, *the system doesn’t work resulting to documentation on papers which is likely tone or lost"* (FGD1).

#### Newborn care guidelines and protocols shortage

Lack of guideline dissemination and their distribution through digital platforms was highlighted as a source of shortage and guideline poor use at the HFs;

“*Currently the Ministry of Health does not distribute or disseminate new guidelines as previously did to inform new updates”* (CM1).

"*Currently guidelines are distributed through a digital platform as an electronic material hence creating a barrier to access by providers*. *Providers prefer printed guidelines*, *however*, *printing facilities at the HFs remain a major concern"* (RM).

#### Best practices to optimize newborn welfare

The study participants reported various initiatives that were locally available to uplift newborn welfare. It included partnerships and social organizations as detailed below: -

#### Partnership and collaboration in addressing challenges related to referral

Participants stressed the importance of inter-institutional relationships is facing scarcity of resources;

"*Our relationship with other institutions has been helpful in the acquisition of an emergency vehicle from nursing school or security agencies for transportation of an emergency newborn case arising when our ambulance has departed elsewhere to transport or pick up another patient"* (CM1).

"*We have an ambulance without an oxygen cylinder*. *When we come across an oxygen-dependent patient*, *we seek assistance from a faith-based hospital whose car is loaded with oxygen cylinders"* (CM4).

Participants at different levels described the importance of an insurance scheme supported by implementing partners (***GIZ under a German Government-Owned Development Bank (KfW)***) in HF acquisition of health commodities and free medical care for mothers and their newborns;

“*There existed an insurance scheme locally known as* ***“Tumaini la mama”*** *which improved maternal and newborn care-seeking as it covered their medical charges"* (FGD1).

"*We had an insurance for pregnant women known as* ***"Tumaini la mama"*** *which was very beneficial*. *The money received from insurance procured medical commodities and maintained equipment*, *however*, *things have changed since the insurance ended"* (CM1).

#### Local grouping geared for newborn survival

At the local level, participants stated two local maternal and newborn health interventions, namely: 1) Wipe my tears, help my mother and me survive, and 2) Labor case notifications for prompt assistance. These interventions aim at tackling the avoidable causes of death to fast-track the reduction of MMs and NMs;

- Wipe my tears, and help my mother and me survive in Swahili (***Nifute machozi*, *tusaidie mimi na mama yangu tuishi***) is a strategy aiming at decelerating MMs and NMs. It reminds everyone in the community to ensure mothers and their newborns survive and thrive by implementing the ***"Jiongeze Tuwavushe Salama***" Campaign. It ensures the welfare of newborns who have survived a maternal death;
  "*The strategy ensures newborns who have survived a maternal death thrive*. *It appeals for clothes*, *money*, *and milk donations from churches*, *mosques*, *individuals*, *and institutions to benefit needy newborns*” (RM).
- Labor case notifications are an initiative to fast-track the management of complicated maternal cases in labor wards. Such cases are being immediately reported to all health administrative levels and a group of experts for immediate technical and non-technical assistance, such as by calling an ambulance, sharing technical expertise, and acquisition of blood to ensure the mother’s survival;

"*Every complicated maternal case in the labor ward is being reported to the managerial and maternal expert groups* [Southern maternity WhatsApp group] *to seek for technical and non-technical support to save mother and newborn lives"* (RM).

"*We’ve improved communication in labor wards*. *When a mother is in labor*, *we track her status*, *and if she develops complications*, *everyone shouts for help to save both lives of the mothers and newborns”* (CM3).

## Discussion

This study assessed HF’s capacity to provide quality IPNC in Mtwara region, Tanzania with a focus on the availability and functionality of NCU/NSUs. Neonatal care and/or stabilization units were unavailable in almost a third of the HFs assessed, or were only partially established and/or running in almost three-quarters of the HFs, which is against the requirement of the Tanzania health policy [29]. As a coping strategy healthcare staff in some HFs admitted newborns to maternity, pediatric, or general in-patient wards, where newborns are mixed with adult patients, hence exposed to more infections [36]. More newborns were being admitted at District hospitals and fewer at health centers, implying that services at the district hospitals need to be enhanced to ensure continuous quality in-patient services to newborns from within the hospitals and those being transferred from lower-level facilities. Although our study shows an increase in the number of HFs with at least any designated NCU/NSU room from 0 in 2010 [37] and 3 in 2014 [38] to 12 or more in 2021 (current study), coverage and performance remain a major concern in HFs in Mtwara region, especially when interpreted in comparison with the national neonatal care guideline [29]. Major gaps in the establishment and running of NCU/NSUs were also distinguished concerning the location of the NCUs/NSUs whereby 25% (3/12) of HFs had their NCUs/NSUs located far from the maternity block indicating a delay in the provision of IPNC during need. This stresses the need to emphasize the requirement for structural design and location of NCUs/NSUs in a way that allows for the appropriate and prompt delivery of in-patient newborn care services.

Although this finding is based in Mtwara region but provides an insight into the availability status of NCUs/NSUs on a much broader scale in the country. Therefore, further studies are recommended in a different setting to establish how far HFs in Tanzania have progressed in the establishment and running of NCUs/NSUs as compared to the 2020 country-wide target of reaching 100% of DHs and 75% of regional and tertiary hospitals with fully functional NCUs [39].

The quantity and quality of in-patient newborn care services offered at the HFs in Mtwara region were also sub-optimal. Nearly a quarter of newborns at risk were not admitted or transferred to NCU/NSUs for diagnosis and management due to limited or unavailable newborn clinical space. Again, the majority of preterm and LBW newborns admitted to NCUs/NSUs did not receive early infancy vaccinations against poliomyelitis (Oral Polio Vaccines), and tuberculosis (Bacille Calmette-Guerin -BCG). This suggests the need for strengthening both obstetric and newborn care services [23] along with improving facilities for assisted feeding and thermal protection at the HFs to serve newborn lives [15].

Focused investments in health workers’ training to scale up skilled attendance at birth, emergency obstetric care as well as neonatal immediate and intensive care is important to reduce gaps in care during the early postnatal period for mothers and babies. Our study documented a shortage of trained healthcare staff at the surveyed HFs, with less than forty percent of the healthcare staff receiving specific newborn care training. As a result, the majority of NCUs/NSUs were staffed by healthcare professionals who had no prior training or orientation in newborn care guidelines and were not receiving ongoing medical training against the standard neonatal care guideline [29]. Also, a document review showed the presence of a lot of unclassified newborn risk factors around a third of newborn cases indicating the provider’s inability of classifying newborn risk factors. The challenges facing health workers in providing quality newborn care in many low and middle-income countries have previously been documented [40, 41]. To improve newborn management toward desired outcomes, institutional structures should ensure on-job training and continuous medical education (CME) programs [42].

Essential medicines such as Tetracycline eye ointment and vitamin K were not available in a majority of HCs and DHs at the time of assessment. This is in contrast to the 2019 national guideline for newborn care and the establishment of NCUs [29], which emphasizes the need of having all essential medicines on hand at all times to ensure timely and effective treatment of needy newborns. Participants during qualitative inquiry pointed out various reasons for the shortages and stock-outs of health commodities including being frequently stocked out at the Tanzania Medical Stores Department (MSD). The shortages of supplies are not uncommon in low and middle-income countries [43, 44]. Sometimes political struggles and processes were pointed to contribute to shortages [45]. This is detrimental in terms of the provision of quality health due to missing items for timely newborn management and is distressful to health care workers [46, 47]. To ensure the ongoing availability of medicines and lifesaving equipment, MSD’s capacity to supply sufficient healthcare commodities to HFs at all times should be reviewed and monitored for supply chain resilience. The use of mobile health (mHealth) combined with other interventions has been proved to serve newborns’ lives and other vulnerable groups [48, 49]. These findings indicate the need for optimizing the supply and management of medicines and consumables along the healthcare continuum.

The presence of banned medicines like phenytoin at some healthcare facilities implies that HCPs are unaware of the national guidelines or are unable to access them. It also implies that regular facility oversight is necessary to address such discrepancies and deficiencies. In addition, most of the consumables were available at the majority of HFs except for the feeding cups and baby diapers. The use of infusion cups as an alternative to feeding cups as stated by the HCPs during qualitative inquiry increases the likelihood of newborn infection. The lack of guideline dissemination and shortage of important newborn care guidelines such as KMC at the majority of HFs as highlighted by participants during qualitative inquiry is due to the current practice of distributing guidelines in form of soft copies (digital guidelines) that need to be reviewed. To cope with digital ways of distributing guidelines, HFs need to be equipped with printing facilities and internet access to enable them with full-time access.

The availability and functioning of life-saving equipment have remained a serious concern, resulting in suboptimal services and/or unnecessary referrals between levels. The inadequacy of cost-effective equipment such as glucometer in the vast majority of HFs 33.3% (4/12) reveals how poorly needy newborns are cared for. Our study supports another study in Bihari India [50] which also reported a comparable disparity between the availability and functionality of equipment in NBCCs, and that nonfunctional equipment was responsible for either sub-optimal or non-use of NBCCs at most healthcare facilities. The barrier to equipment maintenance across public HFs was explained by HCPs as being caused by the shortage of maintenance funds, failure to access biomedical technicians during need, and lack of maintenance sub-contracts. According to a study in Kenya [51], a shortage of middle and highly skilled biomedical equipment maintenance personnel, and relying mainly on self-maintenance personnel, was responsible for poor biomedical equipment maintenance at Kenyatta hospital This situation highlights the need for biomedical technicians’ deployment, decentralization of equipment acquisition, increased financial allocation for equipment maintenance, and improved collaboration between maintenance personnel to ensure equipment maintenance to maintain functionality.

The inability to obtain information on newborn risk factors for 34.8% (1776/4819) newborns as well as the disparity between newborn data reported in HMIS and facility registers during the same reporting period, point to poor recording and reporting that must be watched as it undermines the quality of services and decision making. Data recording and reporting have an impact on evaluating performance indicators that guide decision-making in the direction of quality improvement [16]. Failure to document 28.38% (1022/3600) newborn outcomes stresses the need for continuous capacity building on data management to healthcare providers at all levels of in-patient newborn care through supportive supervision [52]. There absence of proof for newborn death and client opinion reviews to 11 (64.71%) of HFs that reported to be performing regular reviews, implies the need for good leadership at all levels of IPNC to enable informed decision making [53].

Governance considerations in programs are an essential contributor to outcomes in all social determinants of health including education, water, and urbanization as it brings attention to government systems, integration, synergies, citizen engagement, and accountability mechanisms [54]. Priority settings, planning, and funding sources identification for newborns were shown to be major governance drivers for implementation and sustaining programs geared for quality in-patient care. The stakeholders mentioned that the construction of NCU/NSU facilities was possible whenever government priorities were stated clearly and then reflected in the comprehensive council health budgeting and plans (CCHP). However, when funding was inadequate due to poor planning and lack of commitment by managers, it was then a barrier to the establishment of NCU/NSUs in all HFs that provide CEmONC services. In Tanzania, between 2015 and 2019 there has been an improvement in primary HFs in terms of construction, upgrading, and equipping the facilities to provide better and quality health services. Despite the achievements, there are still gaps in the functioning of the upgraded HFs to offer essential maternal and newborn care in the country toward achieving universal health coverage [55].

Participants identified inter-institutional relationships, partnership and collaboration, and public-private partnerships as significant enablers for the establishment and smooth running of NCUs at the HFs. The support from implementing partner was multi-dimensional as it targeted both maternal and newborn health programs in health facilities in terms of procurement and acquisition of health commodities, and health insurance cover for pregnant women delivered mothers and their newborns. In other cases, implementing partners supported getting equipment repaired. Thus, implementing partners helped to overcome bottlenecks related to access to maternal and newborn services, provision of technical assistance, increased funding and investment in newborn health, access to specific commodities and equipment where needed, better data to monitor progress, program improvement, and accountability for results at all levels [56, 57]. Implementing partners add resources and funding that have an impact on child and newborn mortality reduction.

Technical expert support groups for maternal and newborns have the potential to improve survival. The” Southern maternity WhatsApp group” provided a communication platform whereby complicated maternal cases were reported to liaise technical and nontechnical support to save women and newborn lives. Whenever there was any maternal complicated case in the labor ward, immediately was communicated to all health administrative levels and a group of experts through the WhatsApp group. When a report is received, members respond by providing the immediate assistance needed, such as technical expert advice, calling an ambulance, and obtaining blood to ensure the survival of the delivering woman and the newborn. The success and sustainability of such online mentorship and improved communication require robust coordination, technical expertise, demand creation, and financial commitment. Mentorship enhances productivity, and job satisfaction and may ultimately lead to carrier advancement [58]. Thus, exploring the use of e-monitoring could further make technical support available to remote less trained health workers.

The patient’s social and economic factors contributed to delaying care-seeking for neonates leading to poor quality care and poor health outcomes. Most households found it difficult to bear transport costs when given a referral leading to referral refusal and home delivery, then in case of labor complications or giving birth to premature babies, there were major delays to reach hospitals leading to the unfavorable outcome for the mother and newborn. In other cases, a household’s inability to cover caretakers’ living costs make them dislike long hospital stay and prompt them to ask for discharges to leave for home with the unwell baby. The common use of traditional drugs at labor onset was perceived by the community to fasten the labor processes to reduce hospital stay was a detriment as it increased the risk of neonatal and maternal mortality. These factors at the community level affecting the utilization of antenatal care in developing countries have been reported elsewhere [59]. Poor maternal and newborn health awareness contributed to the various life-threatening practices in the community. Thus, awareness creation at the household level using community systems regarding maternal and newborn care and danger signs is necessary and has been shown to improve care-seeking [60, 61].

However, for better performance of community health workers and volunteers, it depends on their relationships with families and the health system support they receive in terms of capacity building and motivation. Improved communication between providers and caretakers of sick newborns admitted to the inpatient care units could increase caretakers’ satisfaction with services and sustain quality care interventions thereafter at home [62, 63]. This translates to a newborn care continuum from facility to the community towards the promotion of family-centered newborn care and shared decisions that are ideal for encouraging community participation.

Community-led strategies have the potential to accelerate the reduction of maternal and newborn mortalities. The “Wipe my tears, help me and my mother survive” initiative in this current study demonstrated teaming up of local health managers and the community at large to provide helping hands for newborns who have survived following cases of maternal mortality. For the needy newborns to thrive, the program calls on stakeholders from churches, mosques, directors, and individual people to donate milk to feed them and other necessities. Socio-economic support for orphaned newborns is necessary due to the lack of opportunity to breastfeed [64] and their increased risk of morbidity and mortality [65], especially in rural areas where poverty is already a problem.

High-quality care for newborns was defined to include the availability of the inpatient newborn care units, and facility readiness in the presence of essential medicines, supplies, and equipment. The presence of trained healthcare workers conversant with newborn care, community members participating in effective care seeking as well as good governance to set priorities for maternal and newborn health and command timely allocation of resources and implementation ensures delivery of IPNC. Effective collaboration through stakeholder engagement was deemed essential to complement government efforts and stimulate actions for newborns and maternal health. Sustaining the various gains observed while working to address the gaps is paramount to attaining high-quality newborn health. Harnessing the global initiatives like the QED [66] and local approaches while working together on an integrated effort to improve quality, equity, and dignity for all women and babies.

While the integration of maternal and newborn care delivery is important, specific newborn health programs need to be prioritized and funded with council administration [67] taking the lead with a health system-wide approach to guide and oversee the implementation, monitoring, evaluation, and learning. Actions to plan and ensure the sustainability of newborn health programs are important through building relationships with stakeholder groups, harnessing powerful champions’ support, and institutionalization of the program within-host routine health systems rather than in parallel implementation [68]. This will improve coverage, access, equity, and efficiency and ensure sustainability and system-wide strengthening for better health of an overall population.

## Study limitations

The survey to HFs was conducted during the COVID -19 peak in 2020. This might have influenced the study findings in one way. Also due to time constraints, we were unable to observe how newborns were being managed in NCUs/NSUs, as a result we focused on previous management records from registers. In view of this, a separate study in this area is recommended especially during the times of COVID -19 free.

## Conclusions

It is therefore concluded from this study that, nearly 88% of the studied HFs in Mtwara were still lagging behind the standard requirement in the establishment and running of NCUs/NSUs. Governance in terms of priority setting and financing, partnership, and collaboration; systemic challenges including poor access to newborn care resources, NCU/NSU facilities, and healthcare staff capacity on specific newborn care; and socio-economic factors like households’ inability to cover referral transport and caretaker hospital living costs and use of traditional drugs were major factors influencing delivery and utilization of quality IPNC services, and the resultant newborn health outcomes. This study recommends the targeted and programmed establishment of NCU/NSUs in all upgraded HFs, to ensure essential medicines, supplies, and lifesaving equipment are available in HFs and from the main supplier (MSD), and recruitment of technicians for equipment maintenance, provision of cascaded specific newborn care training to healthcare staffs, and to collaborate with implementing partners in the provision of technical assistance, focused investment in newborn health, support better data to monitor progress, accountability, and learning. The network of NCUs/NSUs needs to be strengthened and connected with higher-level facilities across the region to ensure synergies and achieve the desired reduction in NMs. This should not only be in Mtwara region, but also in other regions in the country and other places with a similar context in low and middle-income countries to ensure coordinated and sustained provision of quality inpatient newborn care services.
